# SMILE Platform: An Innovative Microfluidic Approach for On-Chip Sample Manipulation and Analysis in Oral Cancer Diagnosis

**DOI:** 10.3390/mi12080885

**Published:** 2021-07-27

**Authors:** Sofia Zoupanou, Annalisa Volpe, Elisabetta Primiceri, Caterina Gaudiuso, Antonio Ancona, Francesco Ferrara, Maria Serena Chiriacò

**Affiliations:** 1 Department of Mathematics & Physics E. de Giorgi, University of Salento, Via Arnesano, 73100 Lecce, Italy; sophia.zoupanou@nanotec.cnr.it; 2 CNR NANOTEC—Institute of Nanotechnology, Via per Monteroni, 73100 Lecce, Italy; elisabetta.primiceri@nanotec.cnr.it; 3 Physics Department, University of Bari “Aldo Moro”, Via Orabona 4, 70126 Bari, Italy; annalisa.volpe@uniba.it (A.V.); caterina.gaudiuso@uniba.it (C.G.); antonio.ancona@uniba.it (A.A.); 4 Institute for Photonics and Nanotechnologies (IFN), National Council of Research of Italy (CNR), 70126 Bari, Italy; 5 STMicroelectronics s.r.l., Via per Monteroni, 73100 Lecce, Italy

**Keywords:** oral cancer, circulating tumor cells, micromixers, 3D microfluidics, biodetection, plastic microfluidics, microfabrication

## Abstract

Oral cancer belongs to the group of head and neck cancers, and, despite its large diffusion, it suffers from low consideration in terms of prevention and early diagnosis. The main objective of the SMILE platform is the development of a low-cost device for oral cancer early screening with features of high sensitivity, specificity, and ease of use, with the aim of reaching a large audience of possible users and realizing real prevention of the disease. To achieve this goal, we realized two microfluidic devices exploiting low-cost materials and processes. They can be used in combination or alone to obtain on-chip sample preparation and/or detection of circulating tumor cells, selected as biomarkers of oral cancer. The realized devices are completely transparent with plug-and-play features, obtained thanks to a highly customized architecture which enables users to easily use them, with potential for a common use among physicians or dentists with minimal training.

## 1. Introduction

Head and neck cancers represent the sixth most common type of cancer in Europe, accounting for 150,000 new patients per year, and 60% of patients with advanced disease at diagnosis die within 5 years [1,2]. The most widespread cancer in the head and neck region is oral squamous cell carcinoma (OSCC), occurring at the border of lips and/or at the posterior of the tongue or palates [3]. Many factors can increase the probability of the disease; tobacco and alcohol consumption [4], oncogenic viruses (e.g., papillomavirus, HPV, or Epstein–Barr virus EBV) [5] and poor oral health [6] are known risk factors. Genetic predisposition is also a key consideration when studying the development of oral cancer. As an example, the role of *NFKB1* gene polymorphisms are currently under investigation [7]. The link of germline genetics and environmental factors to pathologic phenotypes can contribute to a better understanding of the interactive role of the environment, tumor cells, immune cells and microbiome in various diseases [8,9]. Moreover, the importance of correct diet and lifestyle is another crucial aspect in preventing oral cancer, as they also modulate the oral microbiome, which has been demonstrated to play a role in cancer onset, particularly due to its influence in the modulation of immune system [10]. High levels of colonization of OSCC by facultative oral streptococci have been shown [11], and, more recently, Zhang and coworkers compared the microbiota compositions of tumor sites and normal tissues, finding bacteria significantly associated with mouth tumors [12]. These aspects fall in the gene-by-environment (G × E) interaction range, which considers a wider analytical method to study the onset of diseases, including the integration of microbiology into molecular pathology and epidemiology models, going in the direction of more personalized medicine [9].

Currently, biomarkers of oral cancer are indeed inadequate, and inflammatory molecules may have a low specificity. Moreover, the detection of precancerous lesions is not routinely carried out in clinical settings [13]. An easy-to-use, noninvasive assay is strongly needed, and the possibility to perform tests on saliva is an attractive strategy to increase patient compliance [14].

In order to improve the quality of diagnostic and prognostic early screening tests, as well as their availability for a large cohort of potential patients, a number of biomarkers from body fluids have been identified. Among these, inflammatory biomarkers [15,16,17] and circulating tumor cells are the most promising entities to be found in blood or saliva, even at the early stage of the disease, and this method of detection has the potential to be translated into on-chip platforms. Early detection of OSCC is indeed the only way to limit the consequences of disease, and the main challenge in prevention is large-scale screening [18], together with an appropriate diet and correct lifestyle [4]. To reach this goal, the need for a noninvasive assay is compulsory, and the possibility to perform tests on saliva is an attractive strategy to make this test suitable for all, in addition to the ambitious objective of the SMILE platform.

The main purpose of SMILE project is the development of a low-cost sensing device for oral squamous cell carcinoma (OSCC) early screening, with features of high sensitivity, portability, and ease of use, with the aim of reaching a large audience. OSCC is usually diagnosed at an advanced stage, where highly invasive surgery and chemotherapy are required, heavily compromising life quality and survival [3,19].

Breakthrough technologies of the platform include the optimization and integration of a highly customizable plastic microfluidics device able to perform some of the most common operating tools while handling samples, particularly micromixing, gradient generation [20], and the capture and detection of small objects such as circulating tumor cells [21].

Moreover, the recent spread of COVID-19 and its huge impact on clinical settings has forced healthcare systems to undergo a total rearrangement of rules and priorities. In cancer management, this has led to weighing up the risks of tumor progression due to a delay in treatments against the potential of adding to the hospital burden by increasing the risk of exposure to SARS-CoV2. The need of new tools able to maintain standards of diagnosis and control of active tumor cases, while limiting infections, through rapid sample collection is then compulsory. New instruments, based on a lab-on-chip (LoC) approach, with features of low cost and a plug-and-play setup, along with the aim of using body fluids easily collected without the need for healthcare personnel, could revolutionize the approach to periodic screening and follow-ups, allowing the possibility to perform them in a safe and distanced manner.

To meet this goal, microfluidic technologies and the use of plastic substrates in combination with rapid prototyping methods, i.e., fs laser technology and micromilling, seem to be a good alternative to standard methods in the realization of polymeric lab-on-chip, without any constraints on the substrate material [22,23].

Today, several techniques can be exploited for the rapid prototyping of polymeric LoC [24,25]. In particular, thanks to its high resolution (<50 nm), soft lithography is one of the most exploited methods for the rapid prototyping of a polymeric microfluidic device [26,27]. This technology requires the fabrication of a mold, typically by photolithography, as well as replication and assembly of the entire device. Consequently, like other similar molding techniques [28], soft lithography is a time-consuming process, which often limits optimization in prototyping or iterative design. Thus, despite its very low cost, it does not allow an easy and direct translation into large-scale production and industrial exploitation, with the aim of reaching the market of in vitro diagnostics (IVD). A further limitation of soft lithography is the material used. Polydimethylsiloxane (PDMS) microchannels subjected to high liquid pressure undergo deformation [29].

Technologies based on the direct microstructuring of the substrate and, thus, not requiring a mold have been proven to be more suitable during the design of a new device. Among such technologies, three-dimensional (3D) printing gives the possibility of fabricating low-cost 3D microfluidic devices in a single step from a computer model [30,31]. The major concerns about this technique regard the inability to reliably print microfluidic channels with dimensions less than several hundred microns, dimensional fidelity, surface quality, optical transparency, and reduced choice of materials [32]. Conversely, ultrafast laser technology, as a non-clean room process, provides a convenient, economical, and flexible way to fabricate micrometric fluidic patterns by varying the laser parameters [33,34], avoiding the expensive and time-consuming production of masks. The fs laser enables a “cold” ablation of the irradiated volume, which allows the material to be removed by ablation from the irradiated area with negligible thermal damage to the surrounding substrate [35], ensuring high precision and up to submicrometric resolution [36]. Moreover, fs laser pulses do not pose any restriction on the substrate materials [37]. However, the principal constraints of this technique are the high costs of the laser source and them not being efficiently suitable for the fabrication of over-micrometric structures.

Mechanical micromilling is a flexible, cost-efficient, rapid prototyping technology for polymer device machining. In comparison with fs laser, it is more convenient for large features [38]. However, it results in poor surface quality and resolution [39].

The possibility to use plastic devices will significantly improve the reproducibility and stability of experimental setups compared with PDMS-based state-of-art microfluidics. PDMS, despite its features of low cost and disadvantages due to the presence of a hard master obtained by lithographic methods, often suffers from sealing leakage, poor connection stability, consequently low reproducibility of experiments. This aspect is not secondary when dealing with biological methods, as complexity can be achieved only by avoiding variability of the boundary conditions given, i.e., by the device.

Moreover, the possibility to obtain stable connections through customized inlet and outlet holes allows the ease of use necessary to achieve so-called “world-to-chip” connections [40], avoiding the use of magnetic gaskets, clamps, or glue which are usually expensive and time-consuming, and which do not allow reusing the device or capillary tubes [41].

On the other hand, traditional fabrication methods such as lithographical techniques, used to create micro and nanoscale structures, are very useful for prototyping and research experiments, but they do not translate well into mass production, in addition to the use of PDMS, thus limiting its commercial applications owing to the difficulty in upscaling manufacturing and the relatively high cost compared to polymeric alternatives [42]. More critically, intrinsic material properties of native PDMS represent another chapter of drawbacks, such as evaporation, leaching, and absorption of a flowed liquid sample which often makes PDMS unsuitable for repeatable, robust microfluidic biological and chemical analysis applications [43]. Postprocessing of the material (for example, parylene coating) can overcome these limitations but adds an additional backend processing stage and, therefore, makes it more undesirable for commercial manufacturing [44]. Although glass could be used as an alternative, thermoplastics (plastics) are better from a cost and fabrication perspective, allow easy surface treatment, and are generally transparent and biocompatible [45].

The combination of femtosecond laser-based micromanufacturing and micromilling technologies, associated with robust, disposable, plastic substrates and an innovative sealing method and dedicated surface chemical treatment of microchannels for their biological functionalization, has been recently demonstrated for the development of microfluidic devices for biomedical applications. In particular, it has been shown that combining the micrometric precision offered by ultrashort pulsed laser ablation with the higher machining rate of mechanical micromilling is very beneficial for the rapid and flexible prototyping of polymeric lab-on-chips [46]. The as-fabricated devices can be exploited in applications ranging from the simple on-chip study of cells to the onsite and early diagnosis of diseases [47].

The degree of efficiency revealed by the hybrid microfabrication platform proposed in this work would allow producing 3D microfluidic devices, while embedding additional functionalities such as micromixers and gradient generators. This would push the on-chip platform toward the sample in/answer out concept of point-of-care devices.

In the frame of SMILE (SAW-MIP Integrated Device for Oral Cancer Early Detection) project, we explored several aspects of technology, spanning from simulation of the entire platform through finite element methods (FEM) to tests with artificial samples, as well as from a simulation with particle mixing and a gradient generator to the use of microfluidics using oral cancer cells, envisioning the entire on-chip manipulation and analysis of the sample, with a *plug-and-play* device. In this study, two devices, a micromixer developed on two-level microchannels and a serpentine pathway for the biorecognition of circulating tumor cells, which could work separately or in a subsequential manner, were described. The two devices constitute two building blocks toward the realization of a platform including both sample preparation and biodetection. We performed a completely on-chip functionalization of PMMA, demonstrating a step forward with respect to the current literature, as we obtained the desired results without using complex methods such as UV-curable functionalization or low-pressure radiofrequency (RF) air plasma [48,49]. Anti-EpCAM antibody was chosen as a capture probe to distinguish cancer from noncancer cells in a mixture obtained by keeping together oral cancer-derived cells with blood-derived cells [50], thus mimicking the presence of circulating tumor cells (CTCs) in blood samples. In principle, this antibody can be replaced with any other to identify different kind of cells or extracellular vesicles (microvesicles/exosomes), in order to obtain a liquid biopsy from other biological fluids such as saliva or urine. The investigation of CTCs as early biomarkers of cancer is one of the most promising topics in liquid biopsy, and the translation of this research into a point-of-care device is being explored for the high value which an easy-to-use tool could bring in early diagnosis good practices. To this aim, many microfluidic devices for the separation of CTCs and sample enrichment have been realized, including microsieve integration [51,52,53], inertial microfluidics [34], serpentine path, and many other chip architectures. However, in most reported cases, microfabricated microsieves are assembled into PDMS-based lab-on-chips, with the inclusion of a membrane, resulting in a low-exploitable approach. In the case of inertial microfluidics, although demonstrated to be very effective in separation, the technique requires a complex simulation and experimental validation phase [54] to define the right geometry as a function of the properties (e.g., dimensions, Young’s modulus) of the cells to be separated. As the aim of our work was the development of a low-cost easy-to-use device, with the goal of reaching a large audience of possible users and realizing real prevention of the disease, we preferred to use a simple and common serpentine microchannel, in order to lengthen the path that cells are forced to run, with the aim of maximizing the possibility to be captured by immobilized antibodies.

## 2. Materials and Methods

### 2.1. Materials

Both devices were fabricated by assembling different squared layers of transparent PMMA (Vistacryl CQ; Vista Optics, Gorsey Lane, Widnes, Cheshire, UK). Each layer was machined differently according to the design of the device. For the micromixer device, three 30 × 30 mm^2^ PMMA layers were used. The bottom and intermediate layers hosting the micromilled channels were 1 mm thick, while the upper layer containing the inlet/outlet holes was 2.5 mm thick. For the cancer cell capturing device, two square 25 × 25 mm^2^ layers were used. The top layer was 5 mm thick, while the bottom one was 1 mm thick. The bonding between layers was performed with pure isopropyl alcohol (Sigma-Aldrich, St. Louis, MO, USA).

Surface functionalization of the microchannels was required, following a different procedure for each device. Particle mixing required surface passivation, which included O_2_ plasma surface treatment and incubation with 1 mg/mL bovine serum albumin (BSA) (1%) in phosphate-buffered saline (PBS) buffer (Sigma-Aldrich, St. Louis, MO, USA).

The cell capturing, instead, initially included the usage of 3-aminopropyltrirthoxysilane (APTES 5%) in ethanol, glutaraldehyde (0.05%) in water, bovine serum album (BSA) (1%), and Tween^®^-20 (0.05%) in phosphate-buffered saline (PBS). In addition, we used EpCAM mouse monoclonal antibodies (all reagents from Sigma-Aldrich, USA). It is worth emphasizing that the anti-EpCAM antibodies have the advantage of not being reactive with normal or neoplastic nonepithelial cells and recognize only human EpCAM expressed on the surface of the epithelial cells. A secondary labeled antibody anti-mouse IgG (whole molecule)–FITC antibody produced in goat (Sigma-Aldrich, USA) was used for the fluorescence confirmation assay.

The sealing and working principle of both devices was evaluated by performing a series of tests. For the sample injection/pumping and flow control, we used the Elveflow microfluidic setup (Elvesys, Paris, France), suitable for finely tuning flow injection in a range of 0.4–7 µL/min. For real-time acquisition, we used an Axio Zoom V16 fluorescence microscope (Zeiss, Oberkochen Germany), with an ApoZ1x objective and a numerical aperture (NA) of 0.25.

For the micromixers, we carried out two validation tests using colored inks and fluorescent polystyrene microspheres, with diameters of 200 nm (green) and 1 μm (red) (FluoSpheres *^®^* Fluorescent Microspheres, Invitrogen, Ltd. 3 Fountain Drive Inchinnan Business Park, Paisley, UK) in ethanol (Sigma-Aldrich, St. Louis, MO, USA). Instead, for the capturing of tumor cells, we injected cells derived from the OECM-1 human oral squamous carcinoma cell line (purchased from SCC/Sigma-Aldrich) and Jurkatt cell line (leukemic T-cell lymphoblast from ATCC).

### 2.2. Computational Modeling

The micromixing tool was simulated by modeling a 3D h-junction in Comsol Multiphysics 5 (COMSOL, Inc., Burlington, MA, USA), using the Microfluidics module CFD package and, specifically, “mixture model, laminar flow” for predicting the fluid flow and particle transport.

Navier–Stokes equations were used to predict the fluid flow through the 3D network of interconnected channels.

As it can be seen, we established two different microchannels with two independent inlets, sharing a common outlet. As boundary conditions, we set an equal uniform velocity/flow rate on both inlets, and zero pressure was applied at the outlet. Furthermore, from the material library, we selected pure water as the defined fluid to cover all domains inside the network of the channels, and we applied zero-flow conditions at the channel walls. The fluid temperature in the entire simulation was set to 25 °C for water. The fluid was considered incompressible, Newtonian, and with no gravitational effects anywhere in the device. Once the fluid flow regime was parameterized, set and tested, we created a surrogate model for particle mixing. Particle inlets and outlet were arranged following the same logic as the fluidics. Moreover, the applied particle parameters were those of the standard polystyrene beads, using two different sizes of 200 nm and 1 μm, respectively. The drag force was set in accordance with the material’s properties. The type of mesh built for our geometry was free tetrahedral. These conditions were kept constant throughout the simulation. The constructing response was tested for evaluation, after setting all the necessary parameters, by checking the simulation results for the distribution of the flow, velocity, pressure, and particle distribution at all crucial domains of the design.

Selecting the proper channel design with the optimal mixing performance was one of the main steps in optimization. To this end, to prove the quality of our selected model, we examined the flow behavior when experimented with a range of shapes for one of the microchannels, including rectangular, rhomboidal, and elliptical designs for the mixing chamber. The numerical model used was validated by implementing a real system.

### 2.3. Design, Fabrication, and Sealing of PMMA Substrates

**Figure 1:**
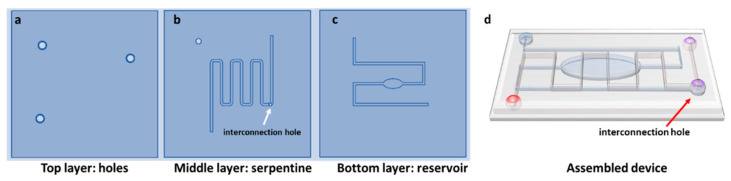
Design of the three layers (**a**) top; (**b**) serpentine; (**c**) bottom and aspects of the assembled device (**d**).

In order to fabricate the microchannels for the mixing module, with desired dimensions of 200 μm width and 200 μm height, and with specific holes as inlet and outlets, we utilized PMMA substrates and the Mini-Mill/GX micromilling machine (Minitech Machinery, Norcross, GA, USA) with a 200 μm two-flute carbide micro end milling tool. The microfluidic network was designed using Solidworks CAD software (SolidWorks Corporation, 300 Baker Avenue, Concord, MA, USA) and transported in machine code file for micromilling control through computer-aided manufacturing (CAM) software. A 150 mm/min feed rate was used to mill the PMMA layers at 20,000 rpm. The alignment of the inlet and outlet with the channels was achieved using an on-board camera of the micromilling machine. The geometry of the micromixer and the aspect of the final assembled device are shown in Figure 1a–d.

The serpentine channel used for the cancer cell capturing experiments was fabricated by exploiting the femtosecond laser milling process, as previously described in [21]. We used an ultrafast solid-state laser system (mod. TruMicro Femto Ed.; TRUMPF GmbH+ Co. KG, Ditzingen, Germany) based on the chirped pulse amplification technique, which delivers linearly polarized 900 fs pulses at a wavelength of 1030 nm with an almost diffraction limited beam (M2~1.3). The laser beam was circularly polarized by a quarter-wave plate and then focused and moved onto the target surface through a galvo-scan head (IntelliSCANNse 14; SCAN-LAB, Puchheim, Germany) equipped with a telecentric lens of 100 mm focal length. The spot diameter at the focal plane was about 25 μm. The fs laser milling process was carried out by removing the material layer by layer, superimposing two perpendicular scanning paths.

The working parameters used for the serpentine channel fabrication are reported in Table 1.

After the fs laser process, loosely attached debris was removed by ultrasonic cleaning in distilled water for 10 min. The dimensions of the fs laser-milled microfeatures were measured using an optical microscope (Nikon Eclipse ME600). Moreover, the average roughness Ra of the milled surface was measured by means of an optical ContourGT InMotion (Bruker, Billerica, MA, USA) profilometer with nanometric resolution and was estimated to be <2 µm. This value is negligible compared to the channel height; therefore, we assumed that the roughness did not affect the fluid flow. The PMMA layer with the fs laser-machined serpentine channel was coupled with a flat and smooth PMMA substrate with inlet and outlet holes drilled using the micromilling machine.

For both the devices, the next step in the fabrication process was the bonding of the PMMA layers. For the assembly of the microfluidic device in both experiments, a thermal- and solvent-assisted bonding method was implemented. In a protected environment we spin-coated hot isopropyl alcohol on the surface of the substrates, aligned the wet slices, and transferred the devices into the oven by holding them in position with clamps and creating an irreversible bonding. In order to build the multilayered chip, it was necessary to reiterate this process twice, i.e., for bonding the substrates with the microchannels, for placing the substrate with the holes on top of the channels. In the end, the channels with the interconnection hole were buried, while the inlets and outlets remained on the upper layer, thus resulting in a monolithic device assembled with no need for additional glue, luer, or gaskets. The two different studies, for mixing of solutions and capturing of CTC, required a diverse functionalization of the microchannels.

The 3D micromixer underwent O_2_ plasma treatment to improve hydrophilicity. After that, the bonding quality and the existence of any leakage were examined by connecting the device to the Elvesys micropumping system through capillary tubes and by gradually increasing the pressure from 10 to 800 mbar. An optical microscope was used for the evaluation. The last step during this process was the in-flow functionalization.

### 2.4. Microchannel Passivation and Functionalization

Although PMMA is a good alternative for customizing the design of microfluidic modules, it suffers from high hydrophobicity. Thus, to attain an optimum functionality of our device, it was essential to mitigate the hydrophobicity of the PMMA surface. To this end, the process could be initiated by treating the assembled PMMA slices with O_2_ plasma, instigating an improved surface wettability and hydrophilicity for an easier flow of water-based solutions. In the subsequent phase, in order to attenuate any sticking of the particles into the channel surface, which could impact the performance, it was necessary to incubate the chip for 2 h with blocking buffer (1 mg/mL BSA in PBS). The sample’s injection during the functionalization process was done directly in-flow. This was mostly obtained thanks to the perfect fitting between the holes and the capillary tubes, allowing for a plug-and-play usage of the device. The stable connections did not require additional glue, gaskets, or clamps to avoid any leakage of the solution, apart from the capillary tubes and the channels.

In the case of the device for cancer cell capturing, it was necessary to flow a sequence of solutions into the microfluidic chip to functionalize it, starting from APTES (5%) in ethanol, in order to increase the hydrophilization and the amine functionality of the PMMA surface. This step was essential both for the easier flowing of the water-based solutions and for promoting the next step. Subsequently, after cleaning the surface with pure water, we injected glutaraldehyde (0.05%) to allow antibody immobilization. Specifically, the glutaraldehyde underwent an imine coupling reaction with the amine group of the antibody, resulting in immobilization of the anti-EpCAM antibody. The ultimate step of this process was the incubation of the device with a blocking buffer (BSA-Tween^®^20 in PBS), to prevent any cell absorption. Moreover, during this process, the device was connected with the micropumping system through a perfect fitting between the capillary tubes and the micromilled inlets.

### 2.5. Experimental Tests for Particles and Coloured Liquids

In order to gain better insight into the performance of the micromixer, the mixing behavior of the device was investigated. Thus, experiments for mixing and gradient generation were performed using colored fluids and particles. We also examined the case when the outlet transmuted into an inlet, to detect the mixing capabilities of the chip and the influence of different paths, lengths, and shapes of the microchannels. Our first attempt was to mix two different colored liquids. For this, we used the capillary tubes to simultaneously inject the liquids into the serpentine and reservoir channels. As a second checkpoint, we chose two different kinds of particles with diameters of 200 nm and 1 μm. Both experiments were performed at different flow rates.

For the injection of both samples, we connected the chip with the Elveflow microfluidic set up. The setup was equipped with an OB1 base module, two MkIII+ channels for pressure control, and two microfluidic sensors, with an analogous temporal flow control. The two inlets were connected with two different vials, containing either the colored liquids or the particles samples. For evaluating the mixing quality, the microchip was placed under a microscope, enabling real-time evaluation of the flow behaviors, as well as image acquisition.

### 2.6. Experimental Tests for Cells

In the CTC capturing experiment, we grew cells in an incubator at 37 °C with 5% CO_2_, in suspension in RPMI 1640 complete growth medium and in adhesion in complete Dulbecco’s modified Eagle’s medium for Jurkat cells and OECM-1 cells, respectively. The growth medium was renewed every 2 days. A few minutes before the experiment started, cancer cells were suspended and harvested in 0.05% trypsin.

For sample injection into the device, we used the Elveflow micropumping system. We visualized the capturing with real-time image acquisition using an Axiozoom Zeiss V16 fluorescence microscope.

Finally, after detachment, the cells were washed and resuspended in DMEM medium. Jurkat cells were centrifuged, counted, and resuspended at the right dilution in order to inject them into the microfluidic chip. Flow rate was tuned at 7 μL/min.

Once the cells remained in contact with the microchannel walls, the microfluidic chip was gently washed with PBS, and adhered cells were stained with a subsequent injection of (i) anti-EpCAM antibody and (ii) secondary FITC-labeled antibody, in order to identify tumor cells blocked at the channels’ surface.

## 3. Results and Discussion

### 3.1. Verification of the Numerical Model

**Figure 2:**
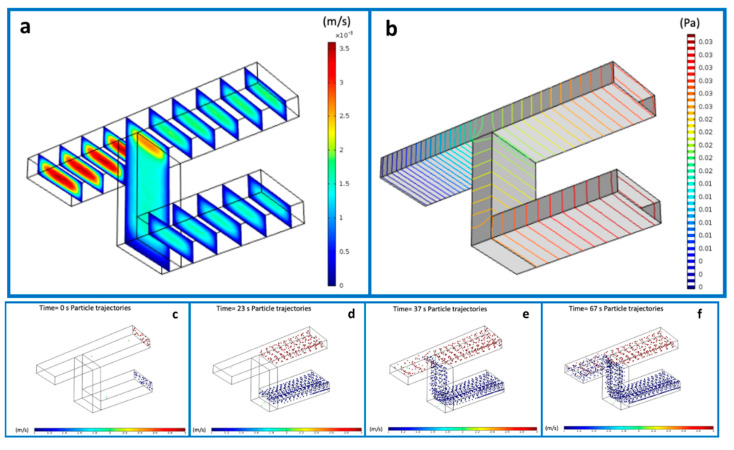
Simulated flow velocity (**a**) and pressure (**b**) in proximity of the interconnection point of the micromixing channel. Mixing of the two populations of nanoparticles at different time points, from the beginning of the experiment (**c**) at time 0 to the complete mixing obtained by (**d**): 23 seconds, (**e**): 37 seconds and accomplished after 67 seconds (**f**).

The 3D fluidic micromixer was studied using CFD simulations, under different regimes, i.e., mixing of fluidics, and mixing and dilution of particles. The results of the interconnected microchannels for the flow velocity distribution and the pressure (Figure 2a,b) offered us the opportunity to gain better insight into the flow behavior. Specifically, Figure 2a depicts the velocity distribution before and after the mixing point, showing a maximal flow at the junction point and the minimal flow predominating at the inlets up to the point of convergence. The velocity at the side walls appeared to be infinitesimal. Concomitantly, the levels of pressure at the entire design are reported in the Figure 2b. As can be seen, the maxima and minima pressure levels were inversely proportional to the velocity. Subsequently, the flow rate was calculated using Equation (1).

Q = Au^−^, (1)

where Q is the flow rate, A indicates the cross-sectional area, and u is the average velocity.

To reveal the particle mixing behavior, a suspension of particles with sizes 1 μm and 200 nm was simulated and injected in both inlets. Moreover, in order to find the best combination of flow rates from the two inlets, we tested a range of values ranging from 1 µL/min to 5 µL/min. The recorded pressure was varied as a consequence of this variation. The mixing of the two different populations is illustrated in Figure 2c–f in the time range of 0–67 s.

**Figure 3:**
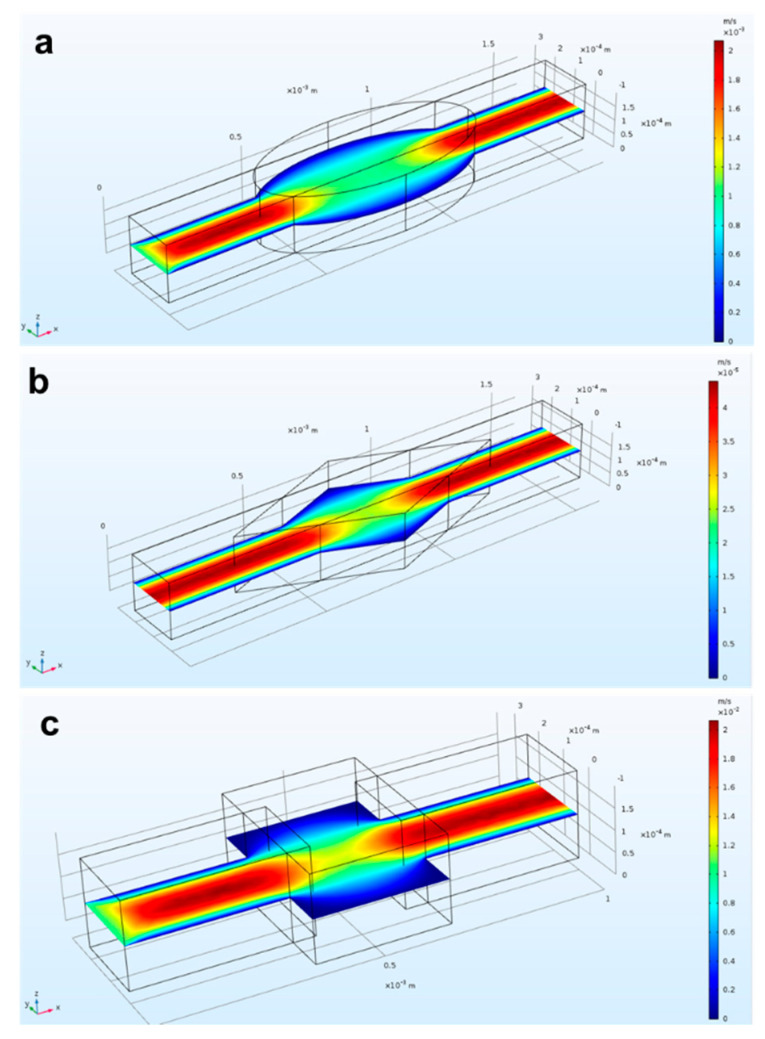
Simulated flow behavior into three different reservoirs: (**a**) rectangle; (**b**) rhombus; (**c**) oval. The lowest flow velocity occurred at the edges of (**a**,**b**).

Design variables, such as the final shape of the microchannels containing a mixing chamber, were investigated. Specifically, we drew and simulated microchannels in rhomboidal, rectangular, and elliptical shapes in order to find the optimal one. Figure 3 shows the comparison of the fluidic velocity for the three different geometries: rectangle (3a), rhombus (3b), and oval (3c). A shared outcome among all cases was the high velocity in the center of the device and lower velocity at the edges. This condition was enhanced for the rectangular shape. Moreover, it was proven from experimental results that angular edges (as can be identified in the rhomboidal and rectangular shapes) are more prone to accumulate bubbles than round edges [55,56]. Thus, in our final device, we incorporated the elliptical shape for realizing the microfluidic reservoir.

### 3.2. Design and Fabrication of the LOC Devices

The findings of our simulation study in predicting the mixing process were verified by fabricating a micromixer with the selected oval and serpentine design. It was of crucial importance to select the optimal design parameters that had the greatest influence on the mixing quality. The first step for the realization of the device was to draw the CAD file and to transfer it into machine code for its fabrication through the micromilling machine. The proposed geometry for mixing experiments, as schematically illustrated in Section 2.3, contained a 7 cm long serpentine-shaped channel in the center of the device organized in six loops, with a reservoir-shaped channel in the bottom with a total length of around 5 cm. The top layer contained three holes (diameter: 1.8 mm) which were defined as inlets and outlets for the capillary tubes. Furthermore, the layer with the serpentine channel also featured a buried hole, which served as a junction point between the channels on the bottom layer and the top layer, thereby creating a 3D microfluidic pathway. The common portion after the junction point ran for 1.5 cm. The device was constructed/assembled from the three individual levels which were separately fabricated, using a mechanical micromilling machine with a 200 μm tool.

**Figure 4:**
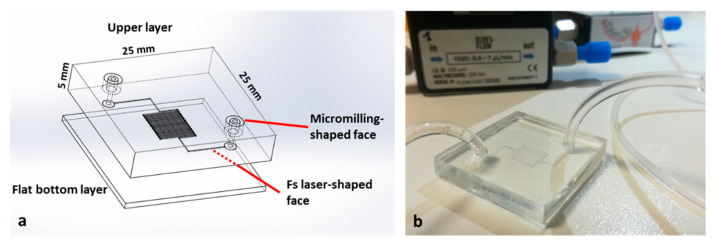
Features of the cell capture device (**a**). PMMA microfabrication allowed for complete transparency of the device. Inlets and outlets were designed to perfectly fit with capillary tubes (**b**).

Regarding the device used for cancer cell capturing, the layout was based on a serpentine microchannel with a square cross-section of 100 μm per side and a total length of 180 mm. The purpose of this design was to increase the active path and proportionally increase the possibility of capturing cells. As displayed in Figure 4a, the device consisted of two PMMA substrates. In this case, the upper substrate was micromilled in both faces. For the lower substrate, we exploited fs laser technology to fabricate the serpentine-shaped channel. To drill the inlet and outlet, we again used a mechanical micromilling machine, this time with a 400 μm tool, ensuring tight connections, since it fit perfectly with the capillary tubes and gave the opportunity for plug-and-play connections. The connection between the holes and the serpentine channel was achieved using two auxiliary channels with a diameter of 600 μm and length of 5 mm, fabricated on the same substrate. Lastly, the bottom PMMA flat layer allowed the sealing of the serpentine channel. No cracks, burrs, or recast layers stemmed from the microfabrication process, thus also providing a great transparency (Figure 4b). Furthermore, the roughness of the bottom channel (Ra = 2 μm) did not affect the fluidic transport of the cells since it was negligible compared to the channel’s height.

The device was assembled and functionalized as described in Section 2.3 and Section 2.4, and the possibility of real-time monitoring of flow into the microchannels, combined with the serpentine shape and a slow flow rate (2 μL/min), enabled us to attain a tool for exploiting a very high surface/volume ratio in terms of active binding sites for antibodies.

### 3.3. Mixing and Gradient Generation Experiments

**Figure 5:**
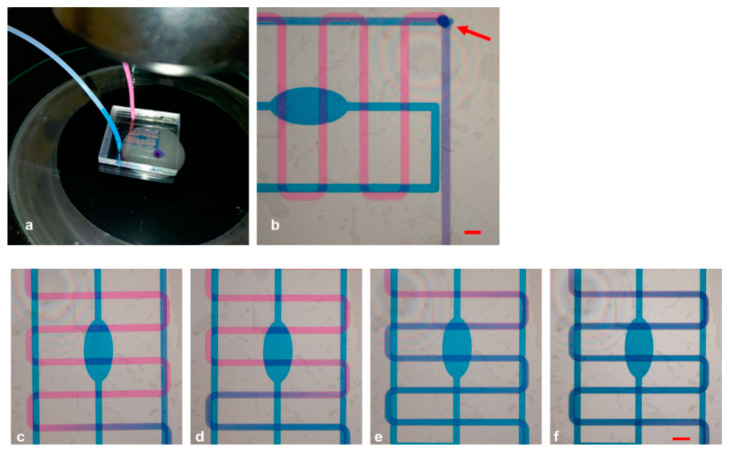
(**a**) Whole device connected to micropumps under the microscope; (**b**) frame acquired after complete mixing of pink and blue ink. (**c**–**f**) Modulation of flow rates, resulting in different mixing conditions of the inks. Scale bars: 500 µm.

We characterized the sealing of the micromixer by visualizing the mixing behavior using two different colored liquids (Figure 5a). The progressive filling of the microchannels was supervised using a microscope. As can be visualized in Figure 5b, the paths of the 3D microchannels could be observed with a single microscope frame, allowing the contemporary monitoring of the multilevel structure. It was of crucial importance to make sure that both solutions arrived simultaneously at the mixing point; hence, we initially set the flow rate at 3 mL/min, but adjusted it later on, when needed. In particular, the complete transparency of the device allowed monitoring the channels while they were progressively filled and differently tuning the parameters due to the diverse shape, resistance, and velocity of the flow in each path. We noticed that the best combination for synchronized arrival at the junction was to set the flow rate to 2.08 μL/min and the pressure to 42.80 mbar for the serpentine channel, whereas these values were set to 1.65 μL/min and 63.08 mbar for the reservoir. The distribution of liquids in the channels and how they initially flowed independently (the pink solution in the upper serpentine channel and the blue one at the reservoir), before being mixed at the meeting point and assuming a violet color, clearly demonstrated that the buried hole (indicated by an arrow) interconnected the two fluids, which became indistinguishable after turning violet. Furthermore, the chip could be used as gradient generator tool using the inlet and outlet alternatively, by tuning the flow rates of the channels and establishing dominance of either the pink or the blue solution (Figure 5c–f).

**Figure 6:**
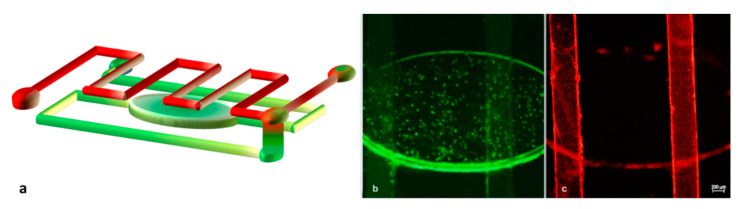
Solutions contained in the two channels flow separately into the device until they reach the interconnection point. (**a**) Scheme of the microchannel network filled with green and red nanoparticles. Green particles run in the bottom channel (**b**), while red ones run in the upper serpentine channel (**c**).

We then assessed the utility of our apparatus using two test samples, where each one contained green fluorescent particles of 200 nm size with 9.1 × 10^5^/mL concentration and red fluorescent particles of 1 μm size and 7.2 × 10^5^/mL concentration, and we injected them into the reservoir and serpentine channel, respectively. The flow of each solution could run separately into the device as explained in the cartoon in Figure 6a. By using the fluorescence microscope, as can be seen in Figure 6b,c, the two different populations flowed separately (the greens were detected only in the bottom channel and the reds were detected only in the upper one). The final injecting parameters we applied in this experiment to simultaneously reach the common portion were a flow rate of 2.13 μL/min and 26.42 mbar pressure for the red particles and a flow rate of 1.46 μL/min and 39.52 mbar pressure for the green particles.

**Figure 7:**
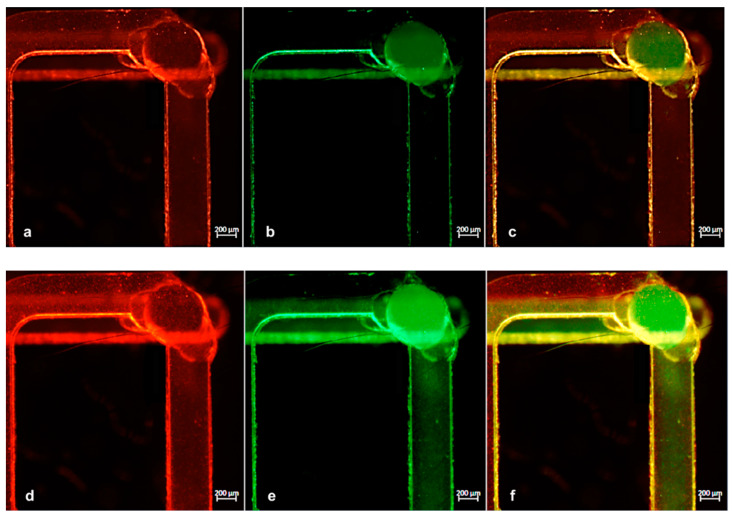
(**a**,**b**) Images of microchannels with green and red particles while running separately before reaching the interconnection point and (**d**,**e**) immediately after. (**c**,**f**) Merged images of green and red fluorescence acquisitions.

Successively, we performed the same experiment using a wide range of flow rates to test the performance of the chip. In all cases, to achieve a mutually proportional flow rate in both channels, every flow change in one channel was followed by a pressure adjustment in the other. Figure 7 presents the time-lapse images of particles captured soon before (Figure 7a–c) and after (Figure 7d–f) the mixing point. Here, the particles moved from their individual channels to the common one, thus verifying the expected mixing efficiency.

### 3.4. CTC Capture Experiments

**Figure 8:**
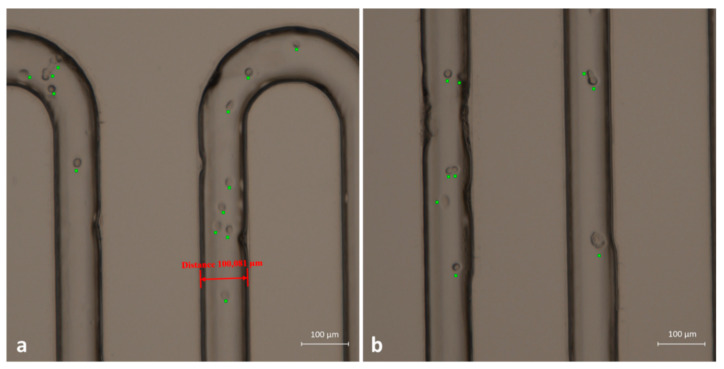
(**a**,**b**) Two frames in bright-field acquisition related to two different regions of the capture device. Attached cells are clearly recognizable and are highlighted by green crosses.

An extensive test was also performed to evaluate the ability of the device to distinguish cancer cells from blood cells. With this aim, we used and immobilized anti-EpCAM antibodies able to recognize human EpCAM, which is a membrane biomarker typically present on the surface of tumoral epithelial cells. The immobilization procedure described in Section 3.3 resulted in the possibility of PMMA microchannels working as capture sites for oral cancer cells. Hence, as a proof of concept, we created a cell mixture composed of two different populations: Jurkat cells (blood-derived cells) and the OECM-1 human oral squamous carcinoma cell line (epithelial-like cells from human oral cancer). In this way, we were able to simulate the contents of a real complex sample. The two different samples were prepared separately and contained 1 × 10^6^ cells/mL from the Jurkat line and 1 × 10^4^ cells/mL from the OECM line. We injected the cell suspensions slowly through the serpentine channel with a flow rate of 7 μL/min. As Figure 8 displays, cells were recognized and blocked as long as they were expressing the EpCAM antigen on their membrane. Therefore, OECM-1 cells were captured on the inner walls of the channels, and most of them remained after the washing with PBS. To maximize the possibility of interaction of cells and wall channels, we used a very low flow rate (2 µL/min) to inject the cell suspension into the serpentine path. Moreover, PMMA device with its complete transparency provided sufficient proof of concept for the successful distinction of cancer cells from normal blood cells and their immobilization in a label-free manner. In the bright-field images of Figure 8, cells fixed to the microchannels walls are highlighted by a green spot.

**Figure 9:**
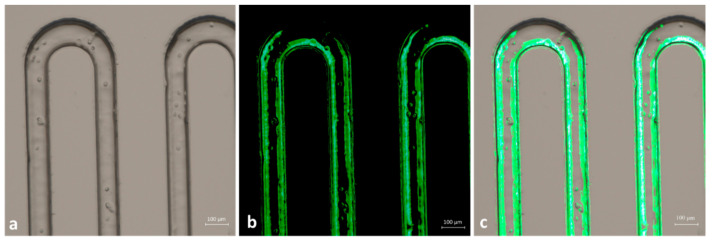
Identification of OECM fixed cells with anti-EpCAM antibody and secondary FITC-labeled antibody. (**a**) Bright-field acquisition of cells; (**b**) green fluorescent acquisition; (**c**) merged image of (**a**,**b**).

In order to demonstrate that captured cells were tumoral cells, we performed some additional labeling. Once the cells were blocked at the microchannels walls, we again injected anti-EpCAM antibody solution, after which we flowed a solution of secondary FITC-labeled antibody, able to bind the Fc portion of the primary antibody. In this way, we were able to selectively label the cell membrane of oral cancer cells. As visible in Figure 9, the membrane of fixed cells was labeled with fluorescent green antibody. We can, thus, conclude that the functionalized microchannels were able to selectively capture tumor cells.

## 4. Conclusions

Plug-and-play devices for the rapid diagnosis of diseases are on the rise for the possibility to obtain quick results in a low-cost and easy-to-use manner. This paper, focusing on the development of a plastic disposable tool, describes the possibility of combining different functionalities, proposing a single chip able to stabilize, preserve, and prepare biological samples. The platform includes a module for sample preparation and mixing of solutions and a detection module, which was demonstrated to capture circulating tumor cells. The produced devices were fabricated via a highly customizable combination of fs laser and micromilling methods using low-cost plastic substrates, while allowing easy connections to the microfluidic system for in-flow functionalization and sample manipulation. The proposed functionalization is a proof of concept which can, in principle, be applied to the detection of other biological entities (exosomes, microvesicles, and so on), by simply modifying the antibody immobilized on the surface of PMMA microchannels. In this case, the validity of the assays was confirmed by using fluorescent probes, which in turn identified the micromixing of nanoparticles and the selective binding of tumor cells in a mixture of normal and cancer cells. The proposed devices may also be of great importance in the case of cancer cell investigations from other body fluids, e.g., saliva, which in turn may require preliminary steps for sample manipulation/dilution or reagent addition. These features, in the era of COVID-19, are very important; for example, a recent release from the American Food and Drug Administration (FDA) authorized the use of home-collected saliva to detect SARS Cov-2. In this way, patients are allowed to self-collect samples for analysis in order to improve accessibility to COVID-19 testing and decrease the risks of infection for medical personnel. Moreover, automatic and low-cost devices, in pandemic contexts, have the possibility to minimize interactions between patients and medical personnel, thus furtherly lowering the probability of infections without affecting access to large-scale screening programs for cancer (and other diseases).

## Figures and Tables

**Table 1:** Laser micromilling parameters.

Repetition Rate (RR)	Pulse Energy	Scan Speed	Hatch Distance
50 kHz	12 μJ	40 mm·s^–1^	5 μm

## Data Availability

Not applicable.
